# Updating the global weighting factor for biodiversity impact assessment in LCA

**DOI:** 10.1016/j.dib.2025.112355

**Published:** 2025-12-03

**Authors:** Julian Quandt, Jan Paul Lindner, Nico Mumm, Barbara von Hippel

**Affiliations:** Institute for Materials Resource Management, Augsburg University, Am Technologiezentrum 8, 86159 Augsburg, Germany

**Keywords:** Life cycle impact assessment, Life cycle assessment, Environmental sustainability assessment, LCA software, Ecoregions, Global weighting

## Abstract

Life‑cycle impact assessment (LCIA) is progressively expanding to address biodiversity impacts, but spatial heterogeneity continues to dominate the associated uncertainty. To account for this, the Ecoregion Factor (EF)—a dimensionless weighting coefficient—modulates biodiversity‑impact scores according to the ecological richness of the ecoregion in which a land‑use takes place. Since the initial publication, the underlying spatial datasets have been superseded by more recent releases. Consequently, we recomputed the EF using the latest ecoregional delineation and the most recent global layers for grasslands, forests and wetlands. To facilitate integration with LCIA frameworks that operate at the national level, the updated EF values were aggregated to country‑level averages by calculating an area‑weighted mean of the normalised EF across all ecoregions intersecting each nation’s borders. This yields a robust geographic adjustment factor that preserves the spatial nuance of ecoregional biodiversity while making biodiversity impact assessments feasible even when inventory data are available only at the country scale. By providing an up‑to‑date, geographically calibrated weighting coefficient, the revised EF enhances the spatial granularity of biodiversity‑focused LCIA results.

Specifications Table

**Table:** 

Subject	Earth & Environmental Sciences
Specific subject area	Updating the global weighting factor for biodiversity impact assessment in LCA
Type of data	Table (.xlsx format), GeoPackage (.gpkg format)
Data collection	Ecoregion factors were derived using QGIS v 3.3 in combination with Excel, following the default formulation proposed by Lindner et al. (2019) [9]. Within QGIS, the mean value of each raster-based input variable was computed for every ecoregion polygon. These zonal means were then exported to Excel, where the final ecoregion factor was calculated. The following datasets were used: Only ecoregions for which all input layers contained valid data were retained; consequently, the raw EF values spanned from 0.037 to 0.509. In line with the procedure described by Lindner et al. (2020) [5], the entire set of factors was then normalized by dividing each value by the minimum EF (0.037). This transformation expresses every factor as a multiple of the lowest-valued ecoregion, yielding normalized EF values ranging from 1 to 13.83. Finally, country-level averages were derived by computing the area-weighted mean of these normalized ecoregion factors across all ecoregions intersecting each nation’s borders.
Data source location	Institution: Institute for Materials Resource Management, University of Augsburg, Germany
Data accessibility	Repository name: Zenodo Data identification number: 10.5281/zenodo.17220373 https://biodiversityvaluemap.mrm.uni-augsburg.de
Related research article	none

## 1 Value of the Data

• **Conceptual foundation** – The Ecoregion Factor (EF) quantifies the ecological value of each ecoregion. The biodiversity damage is scaled by both the intensity of land use and the region’s ecological worth expressed by the EF.
• **Multi-dimensional data basis** – The EF is built from four empirically measurable components that together capture taxonomic, structural, and conservation-priority aspects of biodiversity.
• **Spatial granularity** – The EF enhances the spatial resolution of biodiversity-related LCIA studies. Further, country-aggregated EF values are needed for practical application in LCIA frameworks, since life-cycle inventory data are typically available at the national level.
• **Easy to use** – The EF values are delivered in Excel and shapefiles.

## 2 Background

The rapid decline of biological diversity and the continuing deterioration of ecosystems rank among the most critical issues confronting humanity today [6]. Life Cycle Assessment (LCA) has emerged as a fundamental tool for gauging the environmental performance of products, processes and services. Several models and methods exist to assess biodiversity impacts along the value chain [7]. One of these, the **Biodiversity Value Increment (BVI)**, quantifies the biodiversity quality of the land used by combining multiple biodiversity and land management parameters. This approach was initially presented in Lindner (2016) and further developed in subsequent publications [5,8,9]. A central element of the BVI method is the Ecoregion Factor (EF), which assigns a weight to each ecoregion based on its inherent ecological significance. This weight transforms the generic biodiversity impact per unit area into a location-specific estimate that contextualizes local impacts within a global context.

By using the EF analysts can weight land-use impacts by the ecological importance of the region. Because the underlying datasets are openly accessible, the EF can be updated as better data become available, ensuring long-term relevance. The EF increases the impact scores for land-use activities occurring in high-value ecoregions such as the Central Andean dry puna or the Congo Basin. This weighting steers product-level assessments toward hotspot-aware supply-chain options. The EF delivers a transparent, scalable mechanism to embed biodiversity considerations into LCA workflows.

The present work revises the EF by incorporating current global high-resolution datasets on land cover (Table 1) and adopting the latest terrestrial ecoregion map presented by Dinerstein et al. (2017) [10]. The updated EF values were aggregated to country‑level averages to facilitate integration with LCIA frameworks that operate at the national level. The updated EF values are supplied as .xlsx (data table) and .gpkg (GeoPackage) files.

**Table 1:** Overview of ecoregion factor input indicators, description and source.

Indicator	Description	Source
SGF	Area share of grassland and forest	Copernicus Land Cover 2021 (raster 10 m), global [1]
SW	Area share of wetlands	Ramsar Convention Secretariat [2]
GEP	Global Extinction Probabilities	Kuipers et al. 2019 [3]
SRA	Share of Roadless Areas	Ibisch et al. 2016 [4]

## 3 Data Description

The dataset can be accessed in two ways.

1. **Interactive visualization** – The results can be explored through an interactive web map (Fig. 1) available at https://biodiversityvaluemap.mrm.uni-augsburg.de. The map allows users to view ecoregion factors at both the ecoregion and country level. Several functions enable switching between satellite and schematic map views, searching for specific locations, and centering the map on the user’s position.

**Fig. 1:**
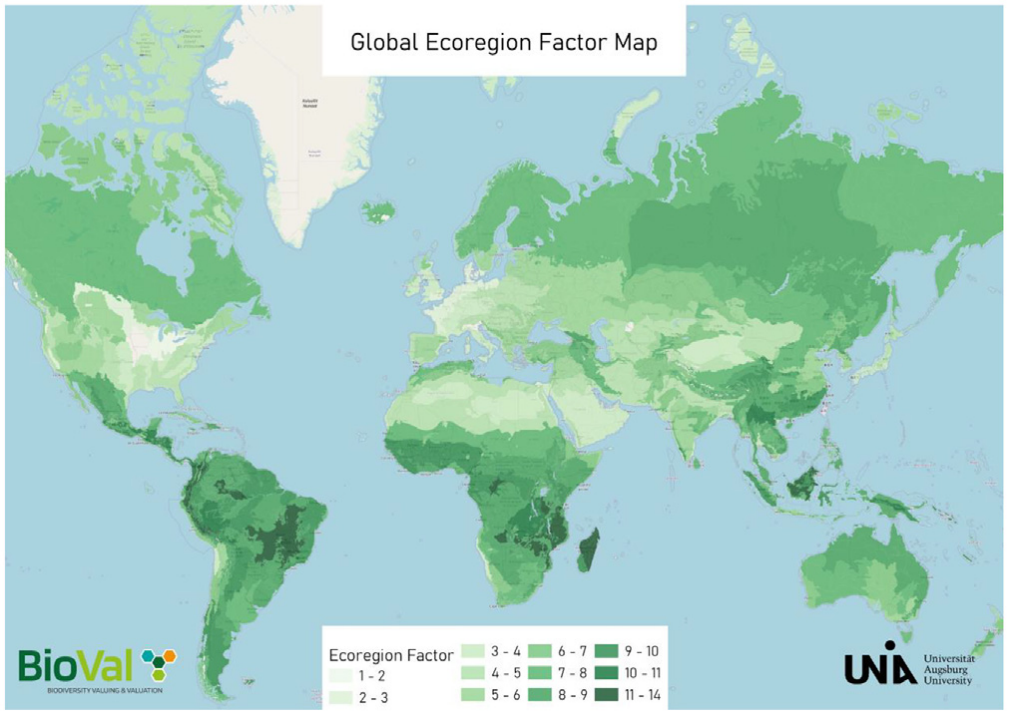
Interactive global web map of ecoregion factors in ecoregion view [13]. Country-level data are also available. Darker green ecoregions represent higher ecoregion factor values, indicating greater global ecological importance than lighter green ones.
2. **Download** – The complete dataset package (Table 2) is deposited on Zenodo (10.5281/zenodo.17220373).

**Table 2:** Content of the downloadable package.

File	Format	Description
ecoregion_factors_v2022_20250926.xlsx	Microsoft Excel workbook	Contains two worksheets: (i) Ecoregion level and (ii) Country level.
ecoregion_factors_v2022_ecoregions.gpkg	GeoPackage	Vector layer with the ecoregion‑level polygons and associated EF values.
ecoregion_factors_v2022_countries.gpkg	GeoPackage	Vector layer with national boundaries and the area‑weighted EF for each country.
  The dataset can be used for further analysis, integration into GIS environments, or large-scale assessments of ecological importance. Researchers may also employ the dataset to combine it with other global indicators, or perform country-specific biodiversity and land-use analyses.

The dataset package comprises the following files:

The file *“ecoregion_factors_v2022_20250926.xlsx”* contains two worksheets i) *Ecoregion level* and (ii) *Country level (*Table 3, Table 4, 4).

**Table 3:** Structure of the Ecoregion level worksheet.

Column	Content
ECO_NAME	Official name of the ecoregion.
BIOME_NAME	Corresponding biome designation.
ECO_ID	Unique identifier for the ecoregion.
EF	Ecoregion Factor calculated in the present study.

**Table 4:** Structure of the Country level worksheet.

Column	Content
COUNTRY	Country name.
ISO_CC	ISO 3166 country codes.
EF	Area‑weighted Ecoregion Factor for the respective country (average of the constituent ecoregion factors, weighted by ecoregion surface within the national boundary).

In the *Ecoregion level* worksheet four columns are provided: (1) ECO_NAME, (2) BIOME_NAME, (3) ECO_ID and (4) EF. A short description of these columns is provided in Table 3.

The *Country level* worksheet provides three columns:(1) COUNTRY, (2) ISO_CC and (3) EF. A short description of these columns is provided in Table 4.

All spatial files use the EPSG:4326 geographic coordinate reference system. The Excel workbook serves as a concise reference table, while the GeoPackage files enable direct import into GIS.

## 4 Experimental Design, Materials and Methods

The ecoregion factor (EF) serves as the global weighting coefficient which separates terrestrial ecoregions worldwide (as defined by Dinerstein et al. (2017) [10]). The ecoregion factor methodology was first introduced by Lindner (2016) and subsequently refined in later publications [5,8,9]. The EF brings local biodiversity impacts into a global context, thus serving as a global weighting mechanism. This enables LCA practitioners to differentiate between land use in ecologically significant regions—such as the Central Andean dry puna or Congo Basin—and less biodiverse areas. The contextualization is achieved through aggregation of four globally available indicators (Table 5): share of grassland and forest (SGF), share of wetlands (SW), global extinction probability (GEP), and share of road-less area (SRA).

**Table 5:** Conceptual mapping of ecoregion factor indicators and CBD biodiversity dimensions.

Indicator	Abbreviation	Description	Data Source, Data Reference Year, Resolution	CBD Biodiversity Dimension	Rationale
Share of Grassland and Forest	SGF	proportion of the ecoregion covered by grassland or forest	[1], 2020, 0.00009° x 0.00009°	Ecosystem Diversity	Represents major terrestrial ecosystem types that support diverse biological communities
Share of Wetlands	SW	proportion of wetland area within the ecoregion	[2], 2023, vector	Ecosystem Diversity	Captures highly productive and biodiverse transitional ecosystems not adequately represented by SGF
Global Extinction Probability	GEP	Aggregated extinction risk for amphibians, birds, reptiles, and mammals based on IUCN Red List status and habitat area	[3], 2016, 0.05° × 0.05°	Species Diversity	Quantifies species-level conservation value and threat status
Share of Roadless Area	SRA	Percentage of area without road infrastructure	[4], 2013, vector	Genetic Diversity	Proxy for habitat integrity and connectivity, which maintain gene flow and evolutionary potential

The selection of these four specific indicators is conceptually aligned with the BVI methodology's fundamental principle of focusing on indicators of biodiversity-supporting conditions rather than direct species counts. This approach is necessitated by the fact that globally, only a fraction of species have been taxonomically described, and comprehensive, spatially explicit species inventories remain largely unavailable at global scales [14]. While alternative indicators such as Red List Indices provide valuable species-level information, their taxonomic coverage is inherently limited and geographically biased toward well-studied regions and taxa [15,16]. In contrast, the selected indicators represent proxies for the underlying conditions that enable high biodiversity to persist.

The SGF and SW indicators represent ecosystem diversity by quantifying the extent of major habitat types. Grasslands, forests, and wetlands constitute the most biodiverse terrestrial ecosystems globally and provide critical ecosystem services [11]. The GEP indicator operationalizes species diversity by integrating IUCN Red List threat assessments with habitat extent, directly reflecting species-level conservation priority. The SRA indicator serves as a proxy for genetic diversity through its representation of habitat connectivity and landscape integrity. Roadless areas maintain gene flow between populations and preserve evolutionary potential [4]. This indicator set balances theoretical comprehensiveness with practical feasibility for LCA applications, avoiding site-specific biodiversity surveys or species-by-species assessments that would exceed the scope and resources available in typical LCA projects.

This approach aims to capture key aspects of the core dimensions of biodiversity —genetic, species, and ecosystem diversity as defined by the Convention on Biological Diversity (CBD) (Table 5) — in a single, reproducible scalar that can be applied across all ecoregions for which data exist. In essence, an ecoregion with a high EF is regarded as more valuable in a global comparison than one with a low EF, and any damage occurring within a high-EF ecoregion is consequently amplified.

The four indicators comprising the ecoregion factor were calculated using spatial analysis and normalization procedures.

The Share of Grassland and Forest (SGF) was calculated by determining the mean areal coverage of grasslands and forests within each ecoregion using zonal statistics applied to global land cover datasets [1]. The grassland and forest shares were summed and normalized. The Share of Wetlands (SW) was calculated by overlaying ecoregion boundaries with the Ramsar Convention wetland database [2] to determine the proportion of each ecoregion occupied by wetlands, which was then normalized.

The Global Extinction Probability (GEP) values for amphibians, birds, reptiles, and mammals were obtained from Kuipers et al. (2019), who calculated extinction probabilities based on species habitat area, IUCN Red List threat status, and regional species richness. For each ecoregion, GEP values across all four taxonomic groups were summed to create a composite extinction risk metric and normalized. Higher GEP values indicate ecoregions harboring more threatened endemic species facing greater extinction risk.

The Share of Roadless Area (SRA) was derived from Ibisch et al. (2016), who mapped roadless areas as contiguous zones beyond 1 km from any road infrastructure. The roadless area fraction for each ecoregion was normalized accordingly.

All indicator values were normalized to the interval [0,^1^], where 0 represents the lowest value and 1 the highest value for each indicator across all ecoregions. The normalized indicator values were then aggregated using Eq. (1) to calculate the ecoregion factor for each of the ecoregions with complete data.

The indicators SGF, SW, GEP and SRA were then aggregated using Eq. (1).

(1) $$\text{EF}=1-\sqrt{\frac{1}{4}*\left({\left(1-SGF\right)}^{2}+{\left(1-SW\right)}^{2}+{\left(1-GEP\right)}^{2}+{\left(1-SRA\right)}^{2}\right)}$$

Equal weighting was applied because: (1) no scientific consensus exists on the relative importance of genetic, species, and ecosystem diversity and (2) the CBD framework treats all three dimensions as equally fundamental.

The root-mean-square aggregation formula is based on a simplification of fuzzy logic principles, which allows for partial fulfillment of multiple criteria without requiring strict thresholds [9,12]. This approach penalizes extremely low values in any indicator reflecting the ecological principle that biodiversity cannot be maintained if any dimension is severely degraded.

The ecoregion factor (EF) could be calculated for 777 of the 847 recognized terrestrial ecoregions. The remaining 70 ecoregions could not be assigned ecoregion factor (EF) values due to insufficient data availability (see supplementary). These data-deficient ecoregions are predominantly small island systems with minimal or negligible agricultural and forestry land use relevant to value chains, such as the Azores temperate mixed forests or Cape Verde Islands dry forests. Due to their limited spatial extent and marginal role in global production systems, these ecoregions are typically of minor significance for LCA applications.

The raw EF scores range from 0.037 to 0.509. Following the procedure outlined by Lindner *et al*. (2020), each raw EF was divided by the minimum value (0.037), converting the set into a dimensionless multiplier that expresses every ecoregion as a multiple of the lowest‑valued one. After this normalisation, the EF range expands to 1 – 13.83.

The minimum-value scaling was chosen to establish the least biodiverse ecoregion as the reference baseline (EF = 1). This approach has both advantages and limitations. (1) LCA characterization factors quantify impacts relative to a reference condition. Setting the lowest-biodiversity ecoregion as EF = 1 enables interpretation of other ecoregions as multiples of this baseline impact potential. (2) The method preserves the full range of variation at the upper end of the distribution, ensuring that exceptionally biodiverse ecoregions (e.g., Madagascar humid forests, EF = 13.83) receive appropriately high weighting factors. (3) It maintains consistency with damage-oriented LCA**:** Higher EF values indicate greater potential biodiversity damage from land use, aligning with the damage-oriented perspective of life cycle impact assessment. By amplifying impact scores for activities in high-value ecoregions, the EF guides supply-chain decisions toward biodiversity-aware sourcing strategies.

The BVI methodology's fundamental principle focusses on indicators of biodiversity-supporting conditions rather than direct species counts. However, using a Spearman correlation test between the combined global Species Richness raster dataset [17] and the Ecoregion Factor, we find a moderate-to-strong positive relationship (ρ = 0.491). The correlation is highly statistically significant (*p* < 2.2 × 10⁻¹⁶), indicating that ecoregions with higher EF values consistently correspond to areas of substantially higher species richness.

Country‑level biodiversity weights were obtained by aggregating the normalised EF values of all ecoregions intersecting a nation’s borders, using an area‑weighted mean to reflect the relative extent of each ecoregion within the country. This approach yielded EF estimates for 210 countries; for the remaining 40 countries, insufficient ecoregional coverage prevented a calculation.

## Limitations

Despite its utility for introducing spatially differentiated biodiversity weighting into life-cycle impact assessment, the EF is constrained by several methodological and data-related shortcomings. First, the factor relies on a limited set of globally aggregated indicators that are available for only 777 of 847 terrestrial ecoregions; consequently, a subset of ecoregions lacks an EF value. Second, the EF treats biodiversity as a homogeneous commodity by aggregating four specific indicators into a single scalar, thereby masking the relative contributions of individual input parameters and precluding insights into whether a low EF value reflects, for instance, limited grassland cover or reduced global extinction probability. Moreover, functional diversity and the distinct ecological roles of different taxa are not captured by this aggregation. Additionally, the fact that three out of four equally-weighted input parameters address habitat diversity might overestimate the contribution of this biodiversity aspect. Third, the EF does not fully account for landscape-level attributes such as connectivity, fragmentation, or patch size, although roadless area serves as a partial proxy for some of these characteristics. Fourth, the EF is static and does not capture temporal dynamics in biodiversity. Fifth, the EF assumes that a unit of land-use impact in a high-EF ecoregion is intrinsically more damaging than the same unit elsewhere, yet local conservation priorities or species vulnerability could modulate actual biodiversity loss. Finally, because the EF is derived from proxy variables rather than direct measures, its validity as a predictor of real-world biodiversity outcomes remains untested.

## Ethics Statement

All authors have read and followed the ethical requirements for publication in Data in Brief and confirm that the current work does not involve human subjects, animal experiments, or any data collected from social media platforms.

## CRediT Author Statement

**Julian Quandt:** Conceptualization, Methodology, Data curation, Writing, Original draft preparation, Reviewing and Editing; **Jan Paul Lindner:** Conceptualization, Methodology, Reviewing and Editing, Funding acquisition; **Nico Mumm:** Methodology, Reviewing and Editing; **Barbara von Hippel:** Reviewing and Editing.

## Data Availability

ZenodoUpdating the global weighting factor for biodiversity impact assessment in LCA (Original data).

## Appendix Supplementary materials

Image, application 1

Image, application 2

Image, application 3
